# Vitamin D screening and targeted supplementation delays liver fibrosis in patients with chronic liver disease: A prospective randomized controlled trial

**DOI:** 10.3389/fmed.2026.1879205

**Published:** 2026-09-29

**Authors:** Changyun Zhong, Wen Yang, Xianrong Yin

**Affiliations:** Shifang People's Hospital, Shifang, Sichuan, China

**Keywords:** 25-hydroxyvitamin D3, chronic liver disease, liver fibrosis, randomized controlled trial, vitamin D

## Abstract

**Objective:**

This study aimed to assess the effects of vitamin D screening and targeted supplementation on the progression of liver fibrosis in patients with chronic liver disease (CLD).

**Methods:**

A total of 60 inpatients with CLD admitted to the Department of Gastroenterology at Shifang People's Hospital between April 2024 and April 2025 were enrolled and randomly assigned to the study group (S group *n* = 30) and the control group (C group *n* = 30). The C group received routine hepatoprotective therapy. The S group received routine therapy plus targeted vitamin D_3_ supplementation based on baseline serum 25-hydroxyvitamin D_3_ [25(OH)D_3_] levels for 12 months. Serum 25(OH)D_3_, aspartate aminotransferase (AST), alanine aminotransferase (ALT), and liver fibrosis markers, such as the AST-to-platelet ratio index (APRI) score and liver stiffness measurement, were assessed at baseline and at 3, 6, and 12 months. Efficacy and safety outcomes were compared between the groups.

**Results:**

At baseline, there were no significant differences in age, gender, disease etiology, disease duration, 25(OH)D_3_ levels, liver function, or fibrosis markers between groups (all *P* > 0.05). After 12 months of intervention, the study group exhibited significantly higher serum 25(OH)D_3_ levels, lower AST and ALT levels, and lower APRI scores and LSMs compared with the C group (all *P* < 0.05). No severe adverse events occurred. Two patients in the study group reported mild gastrointestinal discomfort, which resolved with symptomatic treatment.

**Conclusion:**

Vitamin D screening and personalized supplementation effectively improved vitamin D status in patients with CLD and were associated with favorable reductions in serum liver enzyme levels, in addition to non-invasive surrogate markers of liver fibrosis. Given the limitations of this small single-center unblinded trial, which relied on indirect laboratory and elastographic indicators rather than histological endpoints, large, placebo-controlled studies are required to verify its clinical value before generalized clinical application.

**Trial registration:**

The study was registered in the Chinese Clinical Trial Registry (ChiCTR2600124218).

## 1 Introduction

Chronic liver disease (CLD) is a spectrum of disorders characterized by persistent hepatic inflammation and progressive fibrosis, which can eventually lead to liver failure and hepatocellular carcinoma (HCC) (1). Current therapeutic strategies focus on slowing disease progression and managing complications, but effective antifibrotic interventions remain limited (2). Therefore, identifying safe and accessible interventions to halt or reverse liver fibrosis is clinically urgent.

Vitamin D is a multifunctional fat-soluble vitamin best known for regulating calcium and phosphorus metabolism (3). Accumulating evidence indicates that vitamin D also exerts anti-inflammatory, immunomodulatory, and antifibrotic effects independent of bone metabolism (4, 5). Epidemiological studies have consistently shown that vitamin D deficiency is highly prevalent in patients with CLD and that low vitamin D status is associated with increased liver fibrosis severity (6, 7). Prospective randomized controlled trials investigating the impact of vitamin D supplementation on liver fibrosis are limited, and optimal supplementation regimens remain undefined.

This prospective randomized controlled trial aimed to evaluate the effect of vitamin D screening and targeted supplementation on liver fibrosis in patients with CLD.

## 2 Materials and methods

### 2.1 Study design

Patients with CLD admitted to the Department of Gastroenterology at Shifang People's Hospital between April 2024 and April 2025 were screened.The inclusion criteria were as follows: (1) age ≥18 years; (2) confirmed CLD (chronic hepatitis B, chronic hepatitis C, alcoholic fatty liver disease, compensated cirrhosis, or other chronic liver diseases categorized as “other,” including autoimmune hepatitis and drug-induced chronic liver injury) based on clinical, biochemical, imaging, and etiological evaluations; (3) voluntary participation and signed informed consent; and (4) the ability to complete follow-up. The exclusion criteria were as follows: (1) decompensated cirrhosis, liver failure, HCC, or hepatic encephalopathy; (2) severe cardiac, renal, pulmonary, or endocrine diseases; (3) use of vitamin D supplementation, glucocorticoids, or immunosuppressants within 3 months; (4) pregnancy or lactation; (5) vitamin D allergy; and (6) mental disorders limiting cooperation.

A total of 63 patients were screened, of whom 3 declined to participate. The remaining 60 eligible patients were randomly assigned to the study group (S group, *n* = 30) and the control group (C group, *n* = 30) using a random number table (Figure 1). The study protocol was approved by the Ethics Committee of Shifang People's Hospital (Approval No. 202452). All participants provided written informed consent. The study was registered in the Chinese Clinical Trial Registry (ChiCTR2600124218).

**Figure 1:**
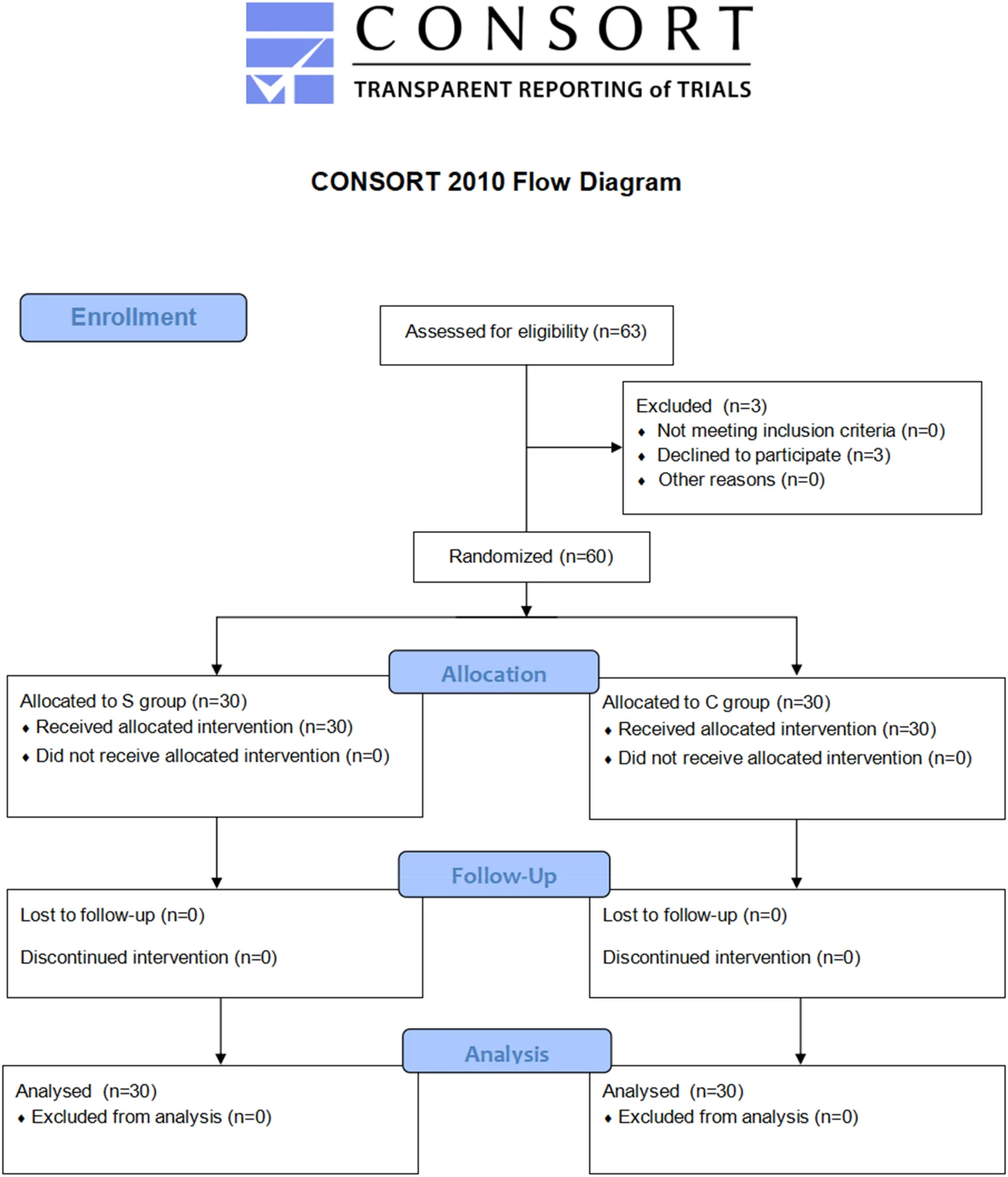
Consort diagram. A total of 63 patients were screened for eligibility; 3 were excluded, and the remaining 60 eligible patients were randomized into two groups (30 patients per group).

### 2.2 Randomization and blinding

Participants were randomly allocated to the study and control groups at a 1:1 ratio using simple randomization, without block randomization or stratification. A statistician, independent of patient recruitment, generated the random sequence using computer software. Sequentially numbered, opaque, sealed envelopes were prepared by a research assistant who did not participate in subject screening and follow-up. At enrollment, a ward research nurse opened the corresponding numbered envelope to confirm group allocation. This trial adopted a single-blind design limited to the outcome assessors only. No matching placebo was provided for vitamin D_3_, so participants, attending physicians, and ward nurses could clearly identify group assignment. Independent laboratory technicians and ultrasound operators who measured liver stiffness measurement (LSM) and analyzed all serum biomarkers remained fully blinded to group allocation during testing and data recording to avoid assessment bias. Staff responsible for randomization, drug administration, and routine clinical management had access to group allocation information.

### 2.3 Intervention

#### 2.3.1 C group

All subjects received a unified 12-month routine therapy comprising polyene phosphatidylcholine (456 mg three times daily) and silybin (140 mg twice daily). Hepatitis B Virus (HBV) patients received entecavir (0.5 mg once daily), whereas Hepatitis C Virus (HCV) patients received genotype-adapted Direct-Acting Antivirals (DAAs). Patients with alcoholic liver disease maintained complete alcohol abstinence. All participants followed a low-fat diet, engaged in regular exercise, and avoided hepatotoxic drugs. No vitamin D supplements were given.

#### 2.3.2 S group

Based on the treatment of the C group, routine therapy plus targeted vitamin D_3_ supplementation was administered according to baseline serum 25(OH)D_3_ levels: deficiency [25(OH)D_3_ < 20 ng/mL], 800 IU/day; insufficiency (20–29.9 ng/mL), 400 IU/day; and sufficiency (≥30 ng/mL), no supplementation, regular monitoring. Doses were adjusted every 3 months to maintain serum 25(OH)D_3_ ≥ 30 ng/mL. The intervention lasted 12 months.

### 2.4 Outcome measures

The co-primary outcomes included the AST-to-platelet ratio index (APRI) and LSM. The secondary outcomes were 25(OH)D_3_, aspartate aminotransferase (AST), and alanine aminotransferase (ALT). Serum 25(OH)D_3_, AST, ALT, the APRI score, and LSM were assessed at baseline (T1), 3 months (T2), 6 months (T3), and 12 months (T4). Serum 25(OH)D_3_ levels were detected using a clinical laboratory assay. Liver function parameters (AST and ALT) were measured using an automatic biochemical analyzer. The APRI score was calculated as follows: (AST/upper limit of normal × 100)/platelet count (10^9^/L). LSM was performed using transient elastography (Mindray R8S). All patients fasted for at least 8 h before detection. Examinations were completed by two ultrasound physicians with over 5 years of TE operating experience. A total of 10 valid measurements were performed per patient; measurements with IQR/median LSM ≤30% were considered reliable, and the median value of the qualified results was used for analysis. The safety assessment included adverse events (e.g., gastrointestinal discomfort, dizziness, fatigue, rash) throughout the trial.

### 2.5 Follow-Up

Patients were followed up every 3 months for 12 months via outpatient visits and telephone interviews. Adherence, adverse events, and clinical symptoms were recorded. All participants completed the 12-month follow-up. Adherence to vitamin D supplementation was assessed through quarterly outpatient and telephone follow-up records, supported by serial serum 25(OH)D_3_ measurements. No participants discontinued supplementation due to adverse reactions, and all patients maintained full adherence throughout the trial.

### 2.6 Statistical analysis

We predefined APRI and LSM as the primary endpoints. Since valid effect sizes for liver fibrosis markers were lacking, we used preliminary 25(OH)D_3_ data for sample size calculation. In the preliminary research, the serum 25(OH)D_3_ level in the C group at T2 was 23.04 (SD = 3.91), whereas it was 26.06 (SD = 3.55) in the S group. A two-sample *t*-test showed that the difference was not statistically significant for the time being (*P* = 0.087, Cohen's *d* = −0.8087). According to the preliminary research results, the sample size calculation was calculated based on the serum 25(OH)D_3_ level at T2 as the primary outcome. A significance level *α* = 0.05 and statistical power of 0.8 were adopted for sample-size calculation. According to the formula, the effective sample size was estimated at *N* ≈ 27 per group. Invasive clinical trials often need to recruit more subjects to prevent patients from dropping out during follow-up. Based on experience, it is assumed that 10% of the trials are lost to follow-up, so each group of samples is adjusted up to N* = *N* ÷ (1 − 0.1) ≈ 30.

Data were analyzed using SPSS 26.0. Continuous variables are expressed as mean ± standard deviation. A paired *t*-test was used for within-group comparisons. An independent *t*-test was used for between-group comparisons. Categorical variables were compared using the *χ*^2^ test. *P* < 0.05 was considered statistically significant. We adopted a per-protocol analysis strategy for all statistical comparisons. No participants were lost to follow-up or withdrew from the intervention throughout the 12-month trial. All 60 randomized subjects completed all follow-up examinations; therefore, the per-protocol population was consistent with the intention-to-treat population.

## 3 Results

### 3.1 Baseline characteristics

No significant differences in age, gender, disease etiology, disease duration, body mass index, serum 25(OH)D_3_ level, AST, ALT, APRI score, or LSM were observed between groups at baseline (all *P* > 0.05). Baseline characteristics are summarized in Table 1.

**Table 1:** Comparison of baseline characteristics between the two patient groups.

Variable	C group (*n* = 30)	S group (*n* = 30)	*t*/*χ*^2^	*P* value
Age (years)	53.2 ± 10.1	53.4 ± 10.3	−0.076	0.94
Gender (male patients/female patients)	17/13	7/23	1.607	0.302
Disease duration (years)	6.8 ± 2.3	7.1 ± 2.5	0.62	0.63
BMI (kg/m^2^)	24.1 ± 3.2	23.8 ± 3.5	0.346	0.73
25(OH)D_3_ (ng/mL)	15.9 ± 4.0	13.6 ± 6.0	1.747	0.086
AST (U/L)	91.4 ± 43.8	93.6 ± 68.8	−0.148	0.083
ALT (U/L)	71.2 ± 33.8	79.8 ± 45.3	−0.833	0.408
APRI score	1.6 ± 0.6	1.8 ± 0.9	−1.013	0.315
LSM (kPa)	8.92 ± 1.8	10.0 ± 2.9	−1.765	0.083
Disease type, *n* (%)				
Chronic hepatitis B	14 (46.7)	15 (50.0)	0.067	0.769
Chronic hepatitis C	5 (16.7)	4 (13.3)	0.131	0.718
Alcoholic fatty liver disease	7 (23.3)	6 (20.0)	0.098	0.754
Others	4 (13.3)	5 (16.7)	0.131	0.718

C group, control group; S group, study group; BMI, body mass index; 25(OH)D_3_, 25-hydroxyvitamin D_3_; AST, aspartate aminotransferase; ALT, alanine aminotransferase; APRI, AST-to-platelet ratio index; LSM, liver stiffness measurement.

### 3.2 Serum 25(OH)D_3_ levels

At baseline, serum 25(OH)D_3_ levels were comparable between groups (*P* > 0.05). At 3, 6, and 12 months, the S group had significantly higher 25(OH)D_3_ levels than at baseline and than the C group (all *P* < 0.05). The C group showed a slight increase at 12 months (*P* < 0.05) (Figure 2).

**Figure 2:**
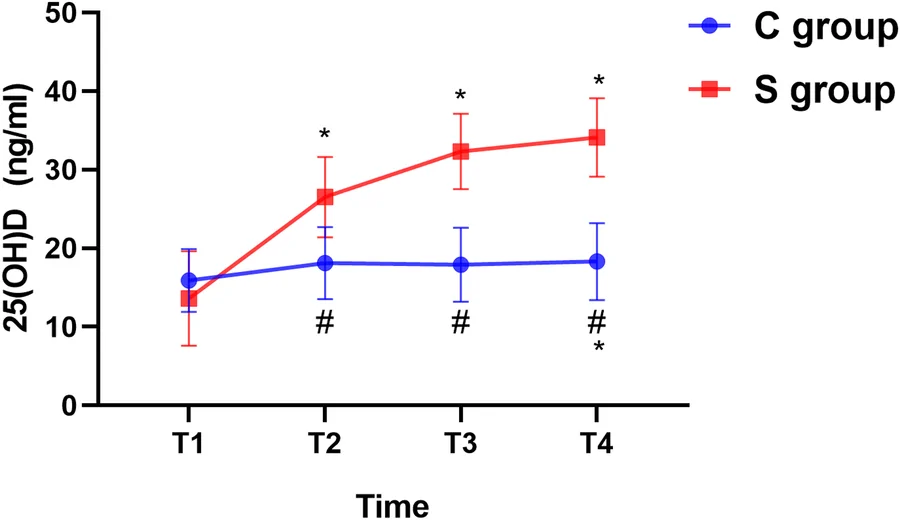
Variables reflecting the 25(OH)D_3_ levels of the two groups at different time points. *n* = 30 for each group. 25(OH)D_3_, 25-hydroxyvitamin D_3_. *P < 0.05 vs. baseline; ^#^*P* < 0.05 vs. control.

### 3.3 Liver function

AST and ALT levels decreased significantly over time in both groups (all *P* < 0.05). The S group exhibited significantly lower AST and ALT levels than the C group at 3, 6, and 12 months (all *P* < 0.05) (Figure 3).

**Figure 3:**
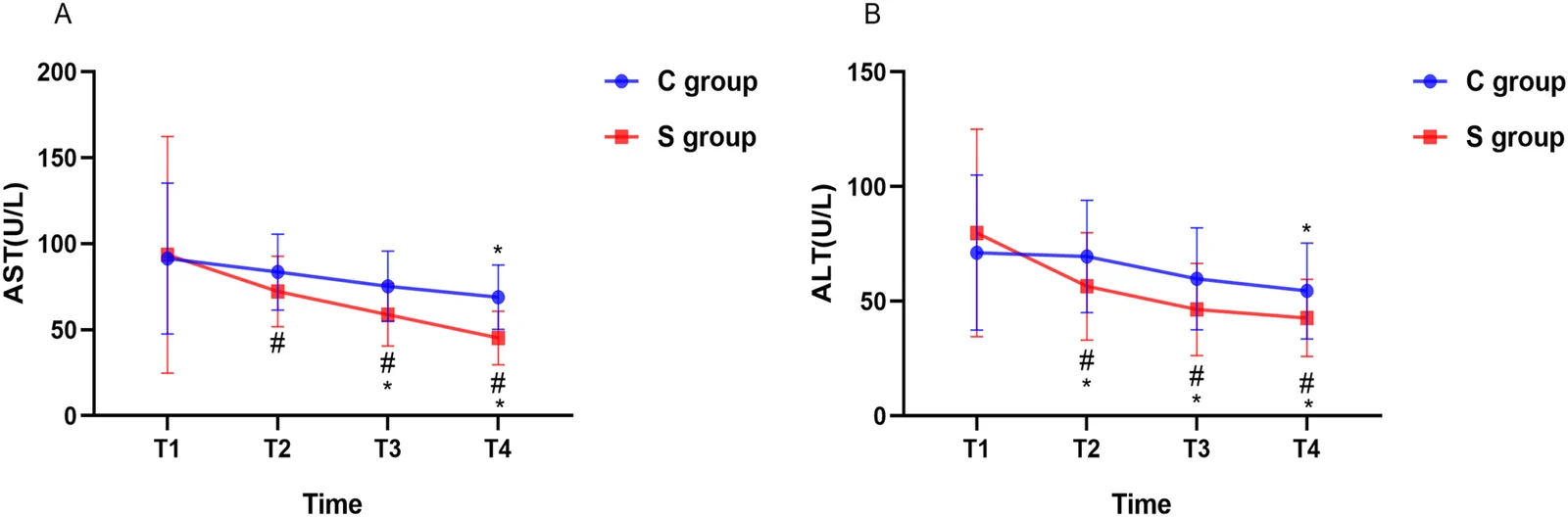
Variables reflecting the AST and ALT levels of the two groups at different time points. *n* = 30 for each group. **(A)** aspartate aminotransferase; **(B)** alanine aminotransferase. *P < 0.05 vs. baseline; ^#^*P* < 0.05 vs. control.

### 3.4 Liver fibrosis markers

The APRI score and LSM decreased significantly in the S group at all follow-up time points compared with those at baseline (all *P* < 0.05). The C group showed a significant reduction in LSM at 6 and 12 months (*P* < 0.05). The S group exhibited significantly lower APRI scores and LSM than the C group at 3, 6, and 12 months (all *P* < 0.05) (Figure 4).

**Figure 4:**
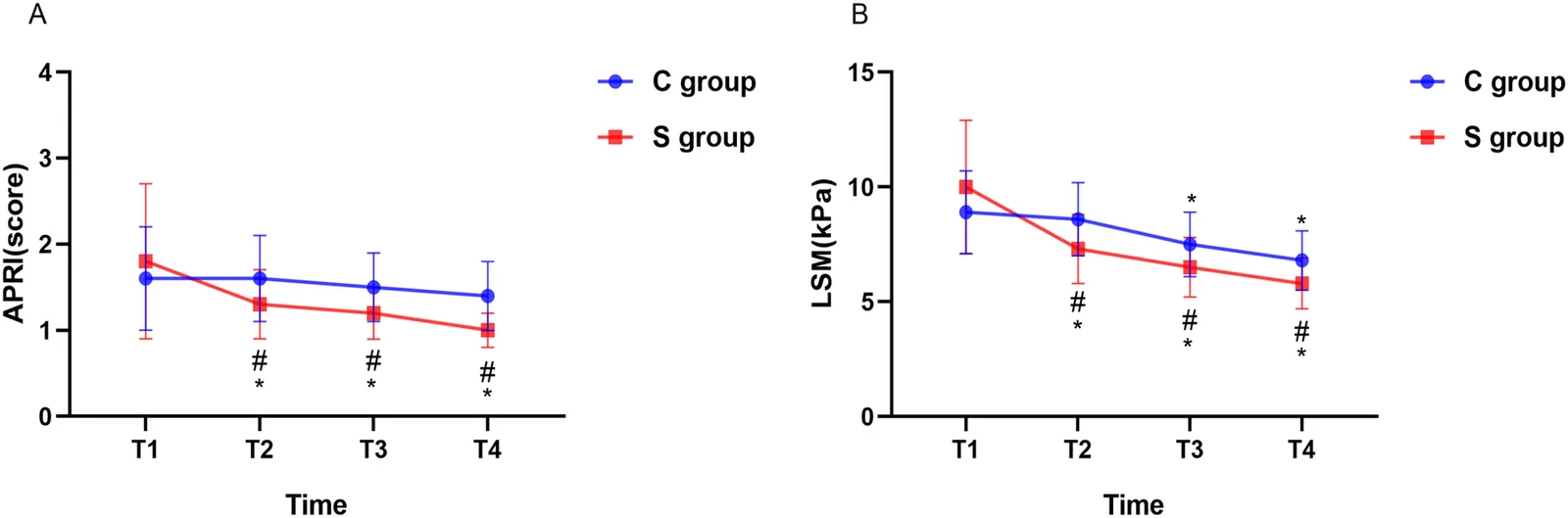
Variables reflecting the APRI and LSM of the two groups at different time points. *n* = 30 for each group. **(A)** AST-to-platelet ratio index; **(B)** liver stiffness measurement. *P < 0.05 vs. baseline; ^#^*P* < 0.05 vs. control.

### 3.5 Safety

No severe adverse events occurred. Two patients in the S group reported mild nausea and bloating, which resolved with post-meal administration. The incidence of adverse events was not significantly different between groups (6.7% vs. 0%, *P* = 0.150).

### 3.6 Follow-Up

All patients completed the 12-month follow-up with 100% adherence.

## 4 Discussion

This prospective, randomized controlled study explored the effects of vitamin D screening and targeted supplementation on liver fibrosis in patients with CLD. The results showed that after 12 months of intervention, the 25(OH)D_3_ levels in the study group were significantly higher than those in the control group, indicating that targeted vitamin D supplementation can effectively correct vitamin D deficiency in CLD patients. Meanwhile, the study group showed markedly lower AST and ALT levels than the control group, suggesting that vitamin D supplementation was associated with improvements in serum liver enzyme profiles in these patients, consistent with findings from previous studies (8). This beneficial effect may be attributed to the anti-inflammatory and antioxidant properties of vitamin D: it modulates the nuclear factor-kappa B (NF-κB) signaling pathway to inhibit the release of pro-inflammatory cytokines such as tumor necrosis factor-alpha (TNF-α) and interleukin-6 (IL-6), thereby mitigating hepatic inflammation (9). In addition, vitamin D enhances mitochondrial function in hepatocytes and reduces oxidative stress-induced damage, ultimately protecting liver cells (10).

The core finding of this study is that vitamin D supplementation was associated with significant reductions in non-invasive surrogate markers for liver fibrosis among CLD patients. Following the intervention, the study group exhibited substantially lower APRI scores and LSM than the control group, with both indicators showing a sustained downward trend. It should be noted that APRI scores and LSM are indirect surrogate markers, susceptible to hepatic inflammation and transaminase changes; the decline in these indicators may partially reflect alleviated liver inflammation rather than definitive suppression or reversal of histological liver fibrosis. These results suggest that vitamin D intervention may help improve fibrosis-related non-invasive markers (11). The underlying mechanisms may involve three key aspects. First, vitamin D inhibits hepatic stellate cell activation—the central effector cells that drive liver fibrosis—because vitamin D binds to vitamin D receptors, thereby suppressing their proliferation, activation, and collagen synthesis and secretion (12). Second, it exerts immunomodulatory effects by modulating the function of T lymphocytes and macrophages in the context of immune dysregulation commonly observed in CLD patients, which mitigates immune-related liver damage and delays fibrosis progression (13). Third, intestinal microecology is optimized, since CLD patients frequently suffer from intestinal dysbiosis, which leads to endotoxin translocation into the circulation that worsens liver damage and fibrosis, whereas vitamin D modulates gut flora equilibrium, diminishes endotoxin uptake, and relieves the liver's workload (14).

Regarding safety, only two patients in the study group developed mild gastrointestinal discomfort that resolved with symptomatic treatment. No severe adverse events occurred, and the incidence of adverse reactions was comparable between the two groups. These findings confirm that vitamin D supplementation is well tolerated and suitable for long-term use. This safety profile aligns with the physiological characteristics of vitamin D. Vitamin D causes few adverse effects within the recommended dosage range. The targeted supplementation regimen in this study, tailored to the patients'’baseline vitamin D levels, avoided the risks associated with excessive intake.

This study has several limitations. First, the study population showed etiological heterogeneity, comprising patients with chronic hepatitis B, chronic hepatitis C, alcoholic fatty liver disease, compensated cirrhosis, and “other” clearly defined conditions (such as autoimmune hepatitis and drug-induced chronic liver injury). These liver diseases vary greatly in disease progression and treatment response, yet we did not perform subgroup analyses stratified by disease etiology. Relevant subgroup exploration will be conducted in subsequent follow-up research. Second, the sample size of this study was relatively small, and there has been no collaboration with other institutions. In the future, we expect to conduct large-scale, multicenter clinical studies. Third, for our four-wave repeated-measure outcome data, statistical comparisons were performed using separate paired- and independent-samples *t*-tests at discrete time points, rather than more robust repeated-measures analysis of variance or linear mixed-effects models. Although there were no missing follow-up data, and all participants completed all study assessments, this analytical approach could not formally evaluate the main effects of group and time nor the critical group-by-time interaction term, simultaneously, which represents an important limitation of the present study. Retrospective re-analysis using mixed-effects models was not performed, given the original pre-specified statistical analysis plan. More rigorous mixed-effects models incorporating covariate adjustment will be implemented in our future prospective trials. Fourth, we did not use placebo capsules for the control group, resulting in unblinded participants and clinicians; however, only outcome evaluators were blinded, which may introduce potential performance bias.

In conclusion, vitamin D screening plus targeted supplementation effectively corrects vitamin D deficiency in patients with CLD. This intervention was associated with improvements in serum liver biochemistry and reductions in non-invasive surrogate markers of liver fibrosis in the present trial. The strategy appears safe, simple, and cost-effective, although further evidence from larger-scale studies is needed to support its clinical implementation. Future work should optimize protocols and develop personalized strategies accounting for individual differences so that more precise, evidence-based guidance can be provided for managing liver fibrosis in patients with CLD.

## Data Availability

The original contributions presented in this study are included in the article/Supplementary Material; further inquiries can be directed to the corresponding author.
